# The Relationship Between Dialysis Patients' Quality of Life and Caregivers' Quality of Life

**DOI:** 10.3389/fphar.2018.00770

**Published:** 2018-07-16

**Authors:** Hiroyuki Nagasawa, Ikuto Sugita, Tomoya Tachi, Hiroki Esaki, Aki Yoshida, Yuta Kanematsu, Yoshihiro Noguchi, Yukio Kobayashi, Etsuko Ichikawa, Teruo Tsuchiya, Hitomi Teramachi

**Affiliations:** ^1^Department of Pharmacy, Secomedic Hospital, Funabashi, Japan; ^2^Laboratory of Clinical Pharmacy, Gifu Pharmaceutical University, Gifu, Japan; ^3^Department of Pharmacy, Chiba Central Medical Center, Chiba, Japan; ^4^Department of Pharmacy, Chuno Kosei Hospital, Gifu, Japan; ^5^Community Health Support and Research Center, Gifu, Japan; ^6^Laboratory of Community Healthcare Pharmacy, Gifu Pharmaceutical University, Gifu, Japan

**Keywords:** dialysis, patient, caregiver, quality of life, multiple logistic regression analysis

## Abstract

Patients on dialysis require caregiving and assistance in their daily lives from family members and/or others for hospital visitation and supervised administration. This places a considerable burden on caregivers, which can in turn influence caregivers' quality of life (QOL). We recruited dialysis patients and their caregivers to elucidate how the QOL of patients relates to that of their caregivers'. Patients completed the EuroQol 5-Dimension scale (EQ-5D) and Kidney Disease Quality of Life-Short Form. Caregivers completed the EQ-5D and Medical Outcomes Study 36-Item Short-Form Health Survey (SF-36). We calculated utility index values for the EQ-5D, and physical, mental (MCS), and role-social component summary scores for the SF-36. Compared to national norms, the caregivers of dialysis patients tended to have poor physical health-related QOL but normal mental health-related QOL, as also found with patients. The multivariate analysis revealed that ≥ median dialysis period and ≥ average burden of kidney disease were significantly related to caregiver MCS score (odds ratios; 6.79 and 9.89, respectively). Caregivers tended to have lower physical health-related QOL if their patients had high social QOL, and lower mental health-related QOL during the early stage of the patient's dialysis treatment, and when patients experienced low disease-targeted QOL.

## Introduction

A large number of persons undergo dialysis treatment: the estimate worldwide was 1.4 million in 2001 (World Health Organization, 2001; Moeller et al., 2002) and 2.6 million in 2010 (Liyanage et al., 2015). In the United States the estimate was 680,000 in 2014 (United States Renal Data System, 2016), and in Japan, over 300,000 in 2011 (The Japanese Society for Dialysis Therapy, 2016). Dialysis requires frequent hospital visits and places restrictions on daily life. As such, these patients often require care and assistance from caregivers. When patients and caregivers are considered together, this reflects a considerable proportion of the population who are involved in the dialysis treatment process.

Dialysis imposes a considerable burden on patients, with hospital visits two or three times per week, and each visit lasting 3–6 h. Dialysis patients have been reported as having low quality of life (QOL) (Yoshiya et al., 2001). Although patients' mental health QOL has been reported to be similar to that of the general population, QOL regarding physical aspects is remarkably low (Erez et al., 2016; Raspovic et al., 2017). Dialysis treatment is a long-term requirement, and family members and/or other caregivers are indispensable for successful continued treatment. However, providing care to dialysis patients is a major burden for caregivers (Belasco and Sesso, 2002; Rioux et al., 2012), who are also reported to have low QOL in certain domains (Belasco and Sesso, 2002; Belasco et al., 2006; Rioux et al., 2012; Santos et al., 2017). Currently, no findings exist regarding the effects, if any, of dialysis patients' QOL on the QOL of their caregivers. Clarification of these effects is essential in ensuring that treatment and care considers not only patients, but also their caregivers. Therefore, in this study we surveyed the QOL of dialysis patients and their caregivers, and conducted multivariate logistic regression analysis to determine the relationship between the two.

## Methods

### Research outlines

QOL questionnaires used in this study were the EuroQol 5-Dimension scale (EQ-5D) (Nishimura et al., 1998), the Medical Outcomes Study 36-Item Short-Form Health Survey v2 (SF-36) (Fukuhara et al., 1998a,b), and the Kidney Disease Quality of Life Short Form version 1.3 (KDQOL-SF) (Green et al., 2001). Both dialysis patients and their caregivers completed EQ-5D to allow for comprehensive measurement of QOL. Caregivers also completed SF-36, a health-profile scale for the comprehensive measurement of QOL, while dialysis patients completed the KDQOL-SF, which is a disease-specific scale and also incorporates the SF-36. As QOL indices, utility values were calculated for the EQ-5D, while for SF-36 and the KDQOL-SF, summary scores were calculated [i.e., the physical component summary (PCS), mental component summary (MCS), and role-social component summary (RCS)]. For stratification of patient attributes, patient QOL, caregiver attributes, and caregiver QOL, analysis was performed on the basis of a set criteria (Table 1).

**Table 1:** Categorizations of patient attributes, patient QOL (quality of life), caregiver attributes, and caregiver QOL.

		**Criteria**	
		**Dichotomous valuable**	
		**1**	**0**
**PATIENT ATTRIBUTES**			
	Patient age	≥65	<65
	Patient gender	F	M
	Education	College, university, or graduate school	Junior high school or high school
	Dialysis period	≥median	<median
**PATIENT QOL**			
EQ-5D	Patient utility index value	≥norm	<norm
KDQOL-SF	Patient PCS	≥50	<50
	Patient MCS	≥50	<50
	Patient RCS	≥50	<50
	Kidney-disease-targeted scales		
	Symptoms/problems	≥mean	<mean
	Effects of kidney disease	≥mean	<mean
	Burden of kidney disease	≥mean	<mean
	Work status	≥mean	<mean
	Cognitive function	≥mean	<mean
	Quality of social interaction	≥mean	<mean
	Sleep	≥mean	<mean
	Non-health-related QOL scales		
	Social support	≥mean	<mean
	Dialysis staff encouragement	≥mean	<mean
	Patient satisfaction	≥mean	<mean
**CAREGIVER ATTRIBUTES**			
	Caregiver age	≥65	<65
	Caregiver gender	F	M
	Education	College, university, or graduate school	Junior high school or high school
**CAREGIVER QOL**			
EQ-5D	Caregiver utility index value	≥norm	<norm
SF-36	Caregiver PCS	≥50	<50
	Caregiver MCS	≥50	<50
	Caregiver RCS	≥50	<50

*MCS, mental component summary; PCS, physical component summary; QOL, quality of life; RCS, role-social component summary*.

### Study design

This was a prospective cross-sectional study using a self-administered questionnaire. Completion of the questionnaire took approximately 10 min and was thus quick and easy for responders to complete.

### Participants

Participants included were 84 pairs of patients receiving dialysis treatment at Secomedic Hospital or Chiba Central Medical Center from June 1 to December 31, 2015, and their caregivers. Patients without caregivers were not included in the study. We asked the patients and their caregivers to participate when they came to the hospitals for dialysis. Patients answered the questionnaire during dialysis, and caregivers answered the questionnaire either during the patient dialysis or at home. Registration of use of EQ-5D (Nishimura et al., 1998), SF-36 (Fukuhara et al., 1998a,b), and KDQOL-SF (Green et al., 2001) was performed prior to study implementation. EQ-5D and SF-36 are used in numerous countries. For SF-36, national standard values (national norms) are published for each country, enabling determinations as to whether a QOL score is higher or lower than the relevant national norm.

### Measures

The survey assessed the following: patient attributes, patient QOL, caregiver attributes, and caregiver QOL.

#### Patients

For patient attributes, we surveyed age, gender, education, and dialysis period. For patient QOL, we used the EQ-5D and the KDQOL-SF.

The EQ-5D is a comprehensive scale that provides a single index value for health status and can be used in health surveys of the general population. The Japanese language version was used in this study. It comprises five dimensions and a visual analog scale (VAS). The five dimensions are mobility, self-care, usual activities, pain/discomfort, and anxiety/depression, each of which are rated on three levels of severity: no problems (level 1), some problems (level 2), and extreme problems (level 3). The EQ-5D index calculator is used to convert the combined scores for each dimension into an overall utility index value with a maximum of 1.000. The VAS evaluates respondents' self-rated health on a vertical scale of 0 to 100 with increments of 10. A score of 0 indicates the worse imaginable health state and a score of 100 indicates the best.

In contrast, the KDQOL-SF measures QOL as affected by kidney disease, rather than overall QOL. It comprises three scales: a kidney disease-targeted scale, non-health-related QOL scale, and generic health-related QOL scale. The kidney disease-targeted scale comprises 40 items arranged in eight subscales: symptoms/problems, effects of kidney disease, burden of kidney disease, work status, cognitive function, quality of social interaction, sexual function, and sleep. The non-health-related QOL scale consists of four items grouped into three subscales: social support, dialysis staff encouragement, and patient satisfaction. All items are rated on a 0–100 scale. Finally, the generic health-related QOL scale contains 36 items that can be categorized into eight subscales: physical functioning, role limitations due to physical health (role-physical), bodily pain, general health, vitality, social functioning, role limitations due to emotional problems (role-emotional), and mental health. The subscale scores range from 0 to 100. Using these subscale scores, norm-based scoring (NBS) can be performed using the national average as the reference. The scores can also be used to calculate three summary scores: PCS, MCS, and RCS. The NBS and summary scores can be converted into population norms based on a mean of 50 and a standard deviation of 10. In general, there are two types of summary scores, with the first consisting of two components (PCS and MCS), and the second consisting of three components (PCS, MCS, and RCS). RCS scores are calculated from three subscales, role-physical, mental health and social functioning. The two-component calculation reflects the factor structure common to Europe and North America, while the three component calculation reflects the factor structure common to Asian countries, including Japan (Fukuhara and Suzukamo, 2015).

#### Caregivers

For caregiver attributes, we surveyed only age, gender, and education. For caregiver QOL, we used the EQ-5D and the SF-36. The SF-36 has the same structure and scoring procedure as the generic health-related QOL scale of the KDQOL-SF.

### Data analysis

Table 1 shows the criteria used for stratification of patient attributes, patient QOL, caregiver attributes, and caregiver QOL. With these criteria, for utility values, persons at or above the national norm (mean value for Japanese persons, i.e., 50) were defined as the high-QOL group, while those below the national norm were the low-QOL group. As for PCS, MCS, and RCS, persons at or above the national norm were defined as the high-QOL group, while those below the national norm were the low-QOL group. The national norm score for utility was 0.877 (Fujikawa et al., 2011) as obtained by Fujikawa et al. in their survey of the general population of Japan. Further, inasmuch as EQ-5D and SF-36 are both scales for comprehensive QOL measurement, when using utility in the analysis, there is no use of PCS, MCS, or RCS; when using PCS, MCS, and RCS in the analysis, utility is not used.

### Statistics

We subjected the stratified data to univariate analysis using Fisher's exact test. We then performed a multivariate logistic regression analysis, including factors that exhibited significance in the univariate analysis at *P* < 0.20 as independent variables, and defining caregiver QOL as the dependent variable. All models were confirmed by adding, in serial order, independent variables beginning with items whose *P* values were lowest in the univariate analysis. We set the significance threshold at *P* < 0.05. The statistical software used was SPSS Statistics 24.

### Ethical considerations

This study was conducted in accordance with the Declaration of Helsinki and the Ethical Guidelines on Biomedical Research Involving Human Subjects. The study was approved by the ethics committees of Gifu Pharmaceutical University (H27-13), Secomedic Hospital (SM2015-27-2), and Chiba Central Medical Center (H27-K2). The patients and their caregivers were given sufficient explanation of the study in writing, and provided written informed consent to participate.

## Results

### Response rate and effective response rate

Of the 84 patient–caregiver pairs surveyed, 51 pairs responded (response rate: 60.7%). The effective response rate was 100%.

### Patient and caregiver attributes

Table 2 shows the results regarding patient and caregiver attributes. The average patient age was 67.7 ± 12.1 (mean±standard deviation), 15 were women (29.4%), and 16 had graduated from college, university, or graduate school (31.4%). The median dialysis period was 85 months. For the caregivers, the average age was 64.5 ± 12.3, 38 were women (74.5%), and 18 had graduated from college, university, or graduate school (35.3%). In terms of carer-patient relationship, 41 pairs (80.4%) were either husband or wife, or an unmarried couple.

**Table 2:** Patient attributes and caregiver attributes.

**(A) PATIENT ATTRIBUTES**	
**Age (no. of years)**	
Mean ± Standard deviation	67.7 ± 12.1
**Gender**	***n (%)***
M	36 (70.6)
F	15 (29.4)
**Education**	
Junior high school or high school	35 (68.6)
College, university, or graduate school	16 (31.4)
**Dialysis period (no. of months)**	
Median (interquartile range)	85 (26–137)
**(B) Caregiver Attributes**	
**Age (no. of years)**	
Mean ± Standard deviation	64.5 ± 12.3
**Gender**	***n (%)***
M	13 (25.5)
F	38 (74.5)
**Education**	
Junior high school or high school	33 (64.7)
College, university, or graduate school	18 (35.3)
**Caregiver-patient relationship**	
Either husband or wife, or an unmarried couple	41 (80.4)
The other relatives	10 (19.6)
The others	0 (0.0)

### Patient QOL and caregiver QOL

#### Patient QOL

Table 3 shows the descriptive statistics for patients' EQ-5D scores and KDQOL-SF summary scores. The response rate for “no problems” (level 1 of severity) was highest (94.1%) for self-care, and lowest (51.0%) for pain/discomfort. The mean patient utility index value was 0.779 ± 0.193 and the average visual analog scale (VAS) health rating was 67.3 ± 19.3.

**Table 3:** Patient EQ-5D scores and patient KDQOL-SF scores.

**(A) PATIENT EQ-5D SCORES**	
	***n* (%)**
**Mobility**	
No problems	30 (58.8)
Some problems	20 (39.2)
Extreme problems (bedridden)	1 (2.0)
**Self-care**	
No problems	48 (94.1)
Some problems	2 (3.9)
Extreme problems (incapable of self-care)	1 (2.0)
**Usual activities**	
No problems	33 (64.7)
Some problems	17 (33.3)
Extreme problems (incapable of usual activities)	1 (2.0)
**Pain/discomfort**	
No problems	26 (51.0)
Some problems (moderate pain/discomfort)	21 (41.2)
Extreme problems (extreme pain/discomfort)	4 (7.8)
**Anxiety/depression**	
No problems	39 (76.5)
Some problems (moderate anxiety/depression)	11 (21.6)
Extreme problems (extreme anxiety/depression)	1 (2.0)
**Patient utility index value**	
Mean ± Standard deviation	0.779 ± 0.193
**Health state**	
Mean ± Standard deviation	67.3 ± 19.3
**(B) PATIENT KDQOL-SF SCORES**	
	**Mean** ± **Standard deviation**
**Summary scores**	
Patient PCS	34.2 ± 15.4
Patient MCS	58.5 ± 9.7
Patient RCS	45.1 ± 17.0
**Generic health-related QOL scales**	
Subscale score (NBS)	
Physical functioning	35.8 ± 19.2
Role physical	35.4 ± 20.8
Bodily pain	50.4 ± 12.2
General health	43.5 ± 11.3
Vitality	52.3 ± 12.6
Social functioning	49.7 ± 12.4
Role emotional	44.3 ± 18.2
Mental health	54.1 ± 11.3
**Disease-targeted scales**	
Kidney-disease-targeted scales
Symptoms/problems	85.4 ± 12.3
Effects of kidney disease	82.4 ± 12.0
Burden of kidney disease	43.3 ± 21.8
Work status	56.9 ± 24.3
Cognitive function	91.2 ± 15.1
Quality of social interaction	92.7 ± 14.8
Sexual function	Not determined
Sleep	70.9 ± 19.6
**Non-health-related QOL scales**	
Social support	79.4 ± 20.2
Dialysis staff encouragement	81.4 ± 16.9
Patient satisfaction	86.9 ± 13.7

*MCS, mental component summary; NBS, norm-based scoring; PCS, physical component summary; QOL, quality of life; RCS, role-social component summary*.

KDQOL-SF summary scores for PCS and RCS (34.2±15.4 and 45.1 ± 17.0, respectively) were below the national norm (50), while the MCS score (58.5 ± 9.7) was above it (Table 3B). For the kidney disease-targeted and non-health-related QOL scales, there were high scores for cognitive function and social interaction (91.2 ± 15.1 and 92.7 ± 14.8, respectively), and low scores for burden of kidney disease (43.3 ± 21.8). We excluded the “sexual function” domain from the analysis because it contained a large amount of missing data.

#### Caregiver QOL

Table 4 shows the results for caregivers' EQ-5D scores and SF-36 scores. In the EQ-5D scores, of all the domains, self-care had the highest rate of “no problems” (level 1) responses (96.1%), while the pain/discomfort domain had the lowest rate (70.6%). The average caregiver utility index value was 0.873 ± 0.160 and the average VAS health rating was 72.9 ± 18.3.

**Table 4:** Caregiver EQ-5D scores and caregiver SF-36 scores.

**(A) CAREGIVER EQ-5D SCORES**	
	******n**** (%)**
**Mobility**	
No problems	40 (78.4)
Some problems	11 (21.6)
Extreme problems (bedridden)	0 (0.0)
**Self-care**	
No problems	49 (96.1)
Some problems	2 (3.9)
Extreme problems (incapable of self-care)	0 (0.0)
**Usual activities**	
No problems	42 (82.4)
Some problems	9 (17.6)
Extreme problems (incapable of usual activities)	0 (0.0)
**Pain/discomfort**	
No problems	36 (70.6)
Some problems (moderate pain/discomfort)	14 (27.5)
Extreme problems (extreme pain/discomfort)	1 (2.0)
**Anxiety/depression**	
No problems	40 (78.4)
Some problems (moderate anxiety/depression)	11 (21.6)
Extreme problems (extreme anxiety/depression)	0 (0.0)
**Caregiver utility index value**	
Mean ± Standard deviation	0.873 ± 0.160
**Health state**	
Mean ± Standard deviation	72.9 ± 18.3
**(B) CAREGIVER SF-36 SCORES**	
	**Mean** ± **Standard deviation**
**Summary scores**	
Caregiver PCS	42.2 ± 13.8
Caregiver MCS	52.8 ± 8.3
Caregiver RCS	51.2 ± 11.7
**Subscale score (NBS)**	
Physical functioning	42.1 ± 16.4
Role physical	47.3 ± 12.4
Bodily pain	49.7 ± 10.6
General health	47.9 ± 9.0
Vitality	50.3 ± 11.5
Social functioning	49.7 ± 12.4
Role emotional	50.2 ± 11.0
Mental health	52.3 ± 10.0

*MCS, mental component summary; NBS, norm-based scoring; PCS, physical component summary; RCS, role-social component summary*.

In the SF-36 scores, the domains of physical functioning (42.1 ± 16.4), role-physical (47.3 ± 12.4), and general health (47.9 ± 9.0) had average scores lower than the norm, while mental health (52.3 ± 10.0) was close to the national average. The caregiver PCS scores (42.2 ± 13.8) were below the national norm, while caregiver MCS scores (52.8 ± 8.3) and caregiver RCS scores (51.2 ± 11.7) were close to the national norm.

### Univariate analysis

Table 5 shows the results of univariate analyses for patient attributes, patient QOL, caregiver attributes and caregiver QOL. For the caregiver utility index value, symptoms/problems (*P* = 0.042), and social support were significantly related (*P* = 0.042). For caregiver PCS, only patient RCS (*P* = 0.046) was significantly related. For caregiver MCS, burden of kidney disease (*P* = 0.016) was significantly related.

**Table 5:** Results of univariate analysis of caregiver QOL.

**(A) CAREGIVER UTILITY INDEX VALUE**			
	**Caregiver utility index value**		***P***
	**<norm (*n* = 21)**	**≥norm (*n* = 30)**	
	***n* (%)**	***n* (%)**	
**Patient attributes**			
Patient age (≥65)	15 (71.4)	19 (63.3)	0.763
Patient gender (F)	4 (19.0)	11 (36.7)	0.221
Patient education (college, university, or graduate school)	8 (38.1)	8 (26.7)	0.541
Dialysis period (≥median)	10 (47.6)	16 (53.3)	0.779
**Patient QOL**			
Patient utility index value (≥norm)	4 (19.0)	14 (46.7)	0.073
Symptoms/problems (≥mean)	9 (42.9)	22 (73.3)	0.042^*^
Effects of kidney disease (≥mean)	10 (47.6)	20 (66.7)	0.249
Burden of kidney disease (≥mean)	8 (38.1)	16 (53.3)	0.394
Work status (≥mean)	3 (14.3)	7 (23.3)	0.495
Cognitive function (≥mean)	14 (66.7)	21 (70.0)	0.529
Quality of social interaction (≥mean)	14 (66.7)	27 (90.0)	0.070
Sleep (≥mean)	8 (38.1)	15 (50.0)	0.568
Social support (≥mean)	9 (42.9)	22 (73.3)	0.042^*^
Dialysis staff encouragement (≥mean)	10 (47.6)	14 (46.7)	1.000
Patient satisfaction (≥mean)	7 (33.3)	12 (40.0)	0.266
**Caregiver attributes**			
Caregiver age (≥65)	13 (61.9)	21 (70.0)	1.000
Caregiver gender (F)	18 (85.7)	20 (66.7)	0.193
Caregiver education (college, university, or graduate school)	8 (38.1)	10 (33.3)	0.772
**(B) CAREGIVER PCS (PHYSICAL COMPONENT SUMMARY)**			
	**Caregiver PCS**		***P***
	<**50 (*****n*** = **33)**	≥**50 (*****n*** = **18)**	
	***n*** **(%)**	***n*** **(%)**	
**Patient attributes**			
Patient age (≥65)	23 (69.7)	11 (61.1)	0.551
Patient gender (F)	10 (30.3)	5 (27.8)	1.000
Patient education (college, university, or graduate school)	12 (36.4)	8 (22.2)	0.358
Dialysis period (≥median)	17 (51.5)	9 (50.0)	1.000
**Patient QOL**			
Patient PCS (≥50)	4 (12.1)	4 (22.2)	0.430
Patient MCS (≥50)	27 (81.8)	15 (83.3)	1.000
Patient RCS (≥50)	21 (63.6)	6 (33.3)	0.046^*^
Symptoms/problems (≥mean)	18 (54.5)	13 (72.2)	0.247
Effects of kidney disease (≥mean)	17 (51.5)	13 (72.2)	0.234
Burden of kidney disease (≥mean)	14 (42.4)	10 (55.6)	0.396
Work status (≥mean)	6 (18.2)	4 (22.2)	0.727
Cognitive function (≥mean)	25 (75.8)	12 (66.7)	0.525
Quality of social interaction (≥mean)	26 (78.8)	15 (83.3)	1.000
Sleep (≥mean)	15 (45.5)	8 (44.4)	1.000
Social support (≥mean)	19 (57.6)	12 (66.7)	0.565
Dialysis staff encouragement (≥mean)	15 (45.5)	9 (50.0)	0.778
Patient satisfaction (≥mean)	14 (42.4)	8 (44.4)	1.000
**Caregiver attributes**			
Caregiver age (≥65)	22 (66.7)	10 (55.6)	0.547
Caregiver gender (F)	25 (75.8)	13 (72.2)	1.000
Caregiver education (college, university, or graduate school)	9 (27.3)	9 (50.0)	0.132
**(C) CAREGIVER MCS (MENTAL COMPONENT SUMMARY)**			
	**Caregiver MCS**		***P***
	**<50 (*n* = 15)**	**≥50 (*n* = 36)**	
	***n* (%)**	***n* (%)**	
**Patient attributes**			
Patient age (≥65)	9 (60.0)	25 (69.4)	0.532
Patient gender (F)	6 (40.0)	9 (25.0)	0.325
Patient education (college, university, or graduate school)	3 (20.0)	13 (36.1)	0.333
Dialysis period (≥median)	5 (33.3)	21 (58.3)	0.093
**Patient qol**			
Patient PCS (≥50)	1 (6.7)	7 (19.4)	0.409
Patient MCS (≥50)	11 (73.3)	31 (86.1)	0.421
Patient RCS (≥50)	7 (46.7)	20 (55.6)	0.759
Symptoms/problems (≥mean)	9 (60.0)	22 (61.1)	1.000
Effects of kidney disease (≥mean)	6 (40.0)	24 (66.7)	0.119
Burden of kidney disease (≥mean)	3 (20.0)	21 (58.3)	0.016^*^
Work status (≥mean)	3 (20.0)	7 (19.4)	1.000
Cognitive function (≥mean)	8 (53.3)	29 (80.6)	0.083
Quality of social interaction (≥mean)	10 (66.7)	31 (86.1)	0.135
Sleep (≥mean)	6 (40.0)	17 (47.2)	0.761
Social support (≥mean)	6 (40.0)	25 (69.4)	0.065
Dialysis staff encouragement (≥mean)	8 (53.3)	16 (44.4)	0.759
Patient satisfaction (≥mean)	6 (40.0)	16 (44.4)	1.000
**Caregiver attributes**			
Caregiver age (≥65)	8 (53.3)	24 (66.7)	0.526
Caregiver gender (F)	10 (66.7)	28 (77.8)	0.487
Caregiver education (college, university, or graduate school)	6 (40.0)	12 (33.3)	0.751
**(D) CAREGIVER RCS (ROLE-SOCIAL COMPONENT SUMMARY)**			
	**Caregiver RCS**		***P***
	<**50 (*****n*** = **20)**	≥**50 (*****n*** = **31)**	
	***n*****(%)**	***n*****(%)**	
**Patient attributes**			
Patient age (≥65)	16 (80.0)	18 (58.1)	0.135
Patient gender (F)	7 (35.0)	8 (25.8)	0.539
Patient education (college, university, or graduate school)	5 (25.0)	11 (35.5)	0.543
Dialysis period (≥median)	10 (50.0)	16 (51.6)	1.000
**Patient qol**			
Patient PCS (≥50)	3 (15.0)	5 (46.1)	1.000
Patient MCS (≥50)	16 (80.0)	26 (83.9)	0.724
Patient RCS (≥50)	10 (50.0)	17 (54.8)	0.780
Symptoms/problems (≥mean)	13 (65.0)	18 (58.1)	0.771
Effects of kidney disease (≥mean)	10 (50.0)	20 (64.5)	0.386
Burden of kidney disease (≥mean)	7 (35.0)	17 (54.8)	0.251
Work status (≥mean)	3 (15.0)	7 (22.6)	0.721
Cognitive function (≥mean)	14 (70.0)	23 (74.2)	0.758
Quality of social interaction (≥mean)	16 (80.0)	25 (80.6)	1.000
Sleep (≥mean)	8 (40.0)	15 (48.4)	0.580
Social support (≥mean)	11 (55.0)	20 (64.5)	0.565
Dialysis staff encouragement (≥mean)	12 (60.0)	12 (38.7)	0.161
Patient satisfaction (≥mean)	8 (40.0)	14 (45.2)	0.778
**Caregiver attributes**			
Caregiver age (≥65)	12 (60.0)	20 (64.5)	0.774
Caregiver gender (F)	13 (65.0)	25 (80.6)	0.324
Caregiver education (college, university, or graduate school)	7 (35.0)	11 (35.5)	0.751

*MCS, mental component summary; PCS, physical component summary; QOL, quality of life; RCS, role-social component summary*,

* *p < 0.05*.

### Multivariate analysis

**Figure 1:**
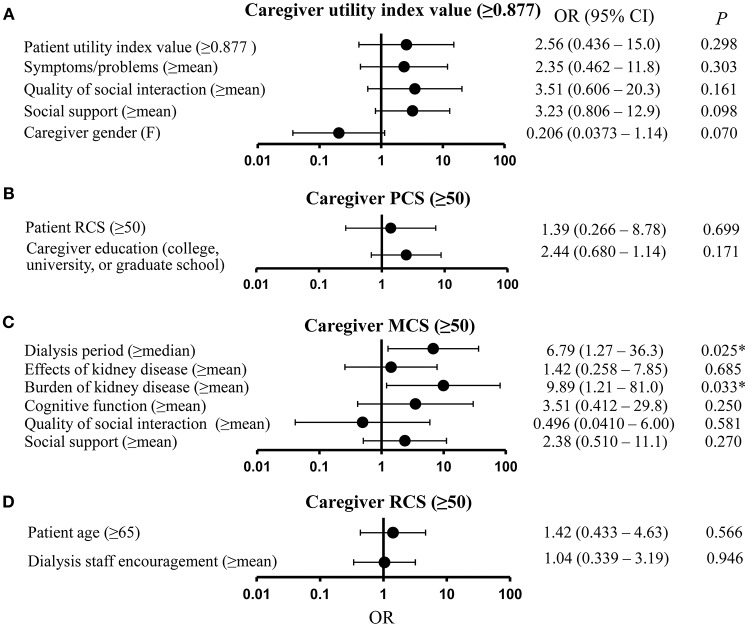
Results of multivariate analysis of caregivers' quality of life (QOL). **(A)** Caregiver utility index value. **(B)** Caregiver physical component summary (PCS) score. **(C)** Caregiver mental component summary (MCS) score. **(D)** Caregiver role-social component summary (RCS) score. CI, confidence interval; MCS, mental component summary; OR, odds ratio; PCS, physical component summary; RCS, role-social component summary, ^*^P < 0.05.

Figure 1A shows the results for the multivariate analysis with caregiver utility index value as the dependent variable and patient utility index value, symptoms/problems, quality of social interaction, social support, and caregiver gender as independent variables, all of which were significant in the univariate analysis at *P* < 0.20. However, none of these variables proved significant in the multivariate analysis.

Figure 1B shows the results for caregiver PCS, and the independent variables of patient RCS and caregiver education, both of which proved significant in the univariate analysis at *P* < 0.20. Neither variable was significant in this analysis. Figure 1C shows the results for the multivariate analysis for caregiver MCS as the dependent variable, and dialysis period, effects of kidney disease, burden of kidney disease, cognitive function, quality of social interaction, and social support as the independent variables. The results showed that having a dialysis period greater than the median (odds ratio [OR] = 6.79; 95% confidence interval [CI] = 1.27–36.3; *P* = 0.025) and a burden of kidney disease greater than the mean (OR = 9.89; 95% CI = 1.21–81.0; *P* = 0.033) were significantly related to caregivers' MCS score. Figure 1D shows the results for caregiver RCS, and the independent variables of patient age and dialysis staff encouragement, both of which proved significant in the univariate analysis at *P* < 0.20. Neither variable was significant in this analysis.

## Discussion

Caregivers of patients on dialysis face a heavy burden (Belasco and Sesso, 2002; Belasco et al., 2006; Rioux et al., 2012; Santos et al., 2017), and minimizing this burden is essential for ensuring that patients can continue treatment in the long term. We measured the QOL of dialysis patients and their caregivers using the EQ-5D, KDQOL-SF, and SF-36, and analyzed the potential relationships between patient and caregiver QOL.

Regarding the patients' EQ-5D results, many patients reported having no problems with self-care, while only a few reported having no problems with pain/discomfort. Thus, although dialysis patients are generally able to care for themselves they continue to struggle with pain and discomfort. The utility index value we obtained was close to the value (0.754) (Katayama et al., 2014) obtained by Katayama et al. but lower than the value (0.877) (Fujikawa et al., 2011) obtained in Fujikawa et al. (2011) survey of the general population.

Regarding patients' KDQOL-SF results, patients had lowest scores for the burden of kidney disease subscale. They also had PCS scores below the national norm. This suggests that dialysis patients experience a heavy burden associated with kidney disease, and have poor physical health-related QOL in general. In contrast, the patient score for MCS was above the national norm, suggesting that they have good mental-health-related QOL. Our findings are consistent with previous studies which have similarly reported that patients with kidney disease have scores below the national standards for burden of kidney disease and PCS, but scores close to national norms for patient MCS (Mazairac et al., 2012; Erez et al., 2016). However, prior research has reported that many dialysis patients suffer depression. Depression constitutes a portion of the mental-health QOL items, depending on the extent of depressive symptoms (Palmer et al., 2013), yet depression does not necessarily entail low patient MCS. While prior reports have found values for patient MCS that are close to the national norm (Mazairac et al., 2012; Erez et al., 2016), in our study, patient MCS was higher, which may be a finding specific to the Japanese sample.

For the EQ-5D results for the caregivers, many reported having no problems with self-care, while only few reported having no problems with pain/discomfort. This finding parallels that for the patients, and suggests that caregivers can care for themselves but tend to struggle with pain and discomfort. Furthermore, the utility index value was close to the reported value (Fujikawa et al., 2011) for the general population in Japan (Fujikawa et al., 2011). Thus, the QOL of caregivers of dialysis patients does not substantially differ from that of the general population.

According to the SF-36 results, caregivers had a score for PCS below the national norm, and a score for MCS which was close to the national norm. These findings suggest that the caregivers of dialysis patients tend to have poor physical health-related QOL but normal mental health-related QOL, as found with patients. Unlike MCS and RCS, PCS begins to decline when a person exceeds age 60 years (Fukuhara and Suzukamo, 2015); yet one cannot conclude from our study results that the physical QOL of dialysis patient caregivers was low. Prior research has reported low scores for caregivers in terms of the role-physical, vitality, and mental health scales for SF-36 (Belasco and Sesso, 2002). Our study differs from prior studies in that caregiver scores for role-physical, vitality, and mental health were close to the national norms.

The results of the multivariate analysis for caregiver utility index value showed that patient attributes, patient QOL, and caregiver attributes were not significantly related to this value. One possible reason for this finding is that the EQ-5D scale has poor sensitivity, owing to its having only three severity levels.

We found that caregivers whose patients had been on dialysis for a long time tended to have higher MCS scores than the general population. One potential reason is that caregivers likely grow accustomed to the patient's dialysis treatment and daily care needs as the treatment period increases. Thus, while caregivers might initially struggle to come to terms with managing patients' treatment, potentially experiencing anxiety and other mental health issues, they may adapt over time. It is also possible that, compared to other diseases, dialysis patients and their caregivers are aware that recovery may also follow from the option of kidney transplant, and so maintain an optimistic outlook. Caregivers whose patients had higher scores for the burden of kidney disease had significantly higher caregiver MCS scores. We found that a low burden of kidney disease entailed a higher mental health QOL of caregivers compared with the general population.

In the multivariate analysis of caregiver PCS and RCS, we found that none of the patient attributes, patient QOL, or caregiver attribute variables were significantly related to caregiver PCS and RCS. This suggests that a high (or low) patient QOL may not impact on the physical and social activities of their caregivers.

In a study of Parkinson's patients and their caregivers, Corallo et al. (2017) found that patient QOL was correlated with caregiver burden (Corallo et al., 2017). Similarly, Borges et al. (2017) reported that caregivers of cancer patients had a greater caregiver burden if patients in their care had low QOL (Borges et al., 2017). Caregiver burden has also been shown to be negatively correlated with the QOL of patients with dementia (Srivastava et al., 2016). However, there are no prior reports on the relationship between dialysis patients' attributes or QOL and the QOL of their caregivers, which was the focus of the current study.

The limitations of this study include the fact that all the dialysis patients we analyzed were on hemodialysis; none were on peritoneal dialysis. The study sample was also small and limited to a specific locality. There are no data on other caregivers' attributes such as caregiver occupation. With regard to the sexual function scale which forms parts of KDQOL-SF, there were many missing data values and so this was excluded from analysis. When the Japanese-language version of KDQOL-SF was under development, there were also many missing data values in surveys of Japanese participants for the sexual function scale, making it difficult to validate (Fukuhara and Suzukamo, 2015). Since each scale is mutually independent, there are no confounding factors or mediators among any of the scales, and inasmuch as sexual acts are not as frequent among Japanese compared with some other countries, it is known that Japanese persons do not place importance on sexual acts in their daily lives (The 2005 Durex Global Sex Survey, 2005). It was thus thought that this specific scale was not of key interest to our current analyses.

## Conclusion

The present study found, that in comparison to the general population, the social QOL of patients was higher, and the physical QOL of their caregivers was lower. Mental health QOL in the early stage of dialysis was lower for patients, and patients experienced a large burden (low QOL) due to their kidney disease; further, the mental health QOL of caregivers was also lower. These results imply that it is possible to improve caregivers' QOL by providing dialysis treatment and care in a way that involves particularly supporting caregivers of socially active patients and those patients who have only recently commenced dialysis treatment (as these caregivers would not yet be accustomed to the treatment), as well as mitigating the burden caused by kidney disease on patients themselves.

## Author contributions

All authors contributed to the study design. All authors participated in collecting and interpreting the data. HN, IS, ToT analyzed data and drafted the manuscript. ToT confirmed the analyzed data and revised the manuscript. All authors reviewed and approved the final manuscript.

### Conflict of interest statement

The authors declare that the research was conducted in the absence of any commercial or financial relationships that could be construed as a potential conflict of interest.
